# Two New Species of *Sidera* (Hymenochaetales, Basidiomycota) from Southwest China

**DOI:** 10.3390/jof8040385

**Published:** 2022-04-10

**Authors:** Zhan-Bo Liu, Meng Zhou, Fang Wu, Jian Yu

**Affiliations:** 1 School of Ecology and Nature Conservation, Beijing Forestry University, Beijing 100083, China; zhanboliu@bjfu.edu.cn (Z.-B.L.); zhoumeng9612@bjfu.edu.cn (M.Z.); 2 Permanent Research Base of National Forestry and Grassland Administration, Jiangsu Vocational College of Agriculture and Forestry, Zhenjiang 212499, China

**Keywords:** phylogenetic analysis, polypore, Rickenellaceae, taxonomy, two new taxa, wood-rotting fungi

## Abstract

Two new wood-inhabiting fungi, *Sidera salmonea* sp. Nov. and *S. tibetica* sp. Nov. in the order Hymenochaetales from southwest China, are described and illustrated based on molecular and morphological evidence. They were found on gymnosperm wood that is rotten and charred. The characteristics of *S. salmonea* include annual, resupinate basidioma, salmon pores with distinctly white margins, angular pores (7–9 per mm), a dimitic hyphal system, and lunate basidiospores that are 3–3.5 × 0.9–1.1 μm. The characteristics of *S. tibetica* include annual, resupinate basidioma with a white to cream fresh pore surface that becomes cream to honey-yellow and shiny when dry, round pores (7–8 per mm), a dimitic hyphal system, and lunate basidiospores that measure 2.9–3.1 × 1–1.1 μm. A phylogenetic analysis based on the combined 2-locus dataset (5.8S + nuclear large subunit RNA (nLSU)) shows that the two species are members of the genus *Sidera*, and they are morphologically compared with related species, respectively. This paper provides a key to the identification of 16 accepted species of *Sidera* that are found throughout the world.

## 1. Introduction

*Sidera* Miettinen &K.H. Larss. is a small genus in the Rickenellaceae Vizzini (Hymenochaetales), which is distributed throughout the world. The genus was typified by *S. lenis* (P. Karst.) Miettinen [1]. The name is derived from *sidus* (star in Latin) and refers to the star-like or rosulate crystals [1]. Thus, the presence of rosette-like crystals is the most striking morphological characteristic of this genus. Its characteristics include resupinate, white to cream or buff fresh basidioma, poroid or hydnoid hymenophores, a monomitic or dimitic hyphal system with generative hyphae that bear clamp connections, the presence of rosette-like crystals, and allantoid to lunate basidiospores [1]. Species in this genus cause a white rot. A total of 14 species to date have been accepted in the genus that is found throughout the world [2].

Six resupinate polypore specimens were collected from the Eastern Himalayas of southwest China during studies on wood-inhabiting fungi, and their morphology corresponded to the concept of *Sidera*. A phylogenetic analysis based on the 5.8S and nuclear large subunit RNA (nLSU) rDNA sequences was conducted to confirm their affinity. Both morphological and molecular evidence demonstrated that these six specimens represent two undescribed species of *Sidera*. Thus, they are described in this paper. In addition, the specimens, literature, and the sequences of all 14 currently accepted taxa of *Sidera* were studied, and their morphological characters are summarized in Table 1. Furthermore, this paper provides an identification key to the accepted species.

## 2. Materials and Methods

### 2.1. Site Description

The type and paratype specimens were collected from eastern Tibet, in southwestern China, ca. E 92°09′, N 26°52′, alt. 3000–3800 m. The vegetation is typical of boreal natural forests and the dominant trees are *Abies georgei*, *Picea linzhiensis* and *Pinus armandii* and *Pinus yunnanensis,* etc.

### 2.2. Morphological Studies

Macro-morphological descriptions were based on voucher specimens and field notes. Microscopic structures were prepared from slide preparations of dried tissues stained with Cotton Blue and Melzer’s reagent, as described by Dai [3]. The following abbreviations are used: CB = Cotton Blue; CB– = acyanophilous in Cotton Blue; IKI = Melzer’s reagent; IKI– = neither amyloid nor dextrinoid in Melzer’s reagent; KOH = 5% potassium hydroxide; n (a/b) = the number of spores (a) measured from a given number of specimens (b); L = mean spore length (arithmetic average of basidiospores); W = mean spore width (arithmetic average of basidiospores); and Q = variation in the L/W ratios between the specimens studied. When the variation in spore size is shown, 5% of the measurements were excluded from each end of the range, and these values are shown in parentheses. Special color terms follow [4] and then herbarium abbreviations [5]. The voucher specimens for the present study are been deposited in the herbarium of the Institute of Microbiology, Beijing Forestry University (BJFC), Beijing, China.

### 2.3. DNA Extraction, PCR, and Sequencing

Total genomic DNA was extracted from dried specimens using a CTAB Rapid Plant Genome Extraction Kit (Aidlab Biotechnologies Company, Ltd., Beijing, China) according to the manufacturer’s instructions with some modifications [6]. The ITS regions were amplified with primers ITS4 and ITS5 [7]. The nLSU regions were amplified with primers LR0R and LR7 (http://www.biology.duke.edu/fungi/mycolab/primers.htm, accessed on 7 March 2022).

The polymerase chain reaction (PCR) procedure for the ITS was as follows: initial denaturation at 95 °C for 3 min, followed by 35 cycles at 94 °C for 40 s, 58 °C for 45 s, 72 °C for 1 min, and a final extension of 72 °C for 10 min. The PCR procedure for the nLSU was as follows: initial denaturation at 94 °C for 1 min, followed by 35 cycles at 94 °C for 30 s, 48 °C for 1 min, and 72 °C for 1.5 min, and a final extension of 72 °C for 10 min [8]. Aliquots of PCR products were examined on 2% agarose gels stained with GelStar Nucleic Acid Gel Stain (Lonza Rockland, Inc., Rockland, YN, USA) and examined under UV light. The sequencing of the PCR products was conducted by the Beijing Genomics Institute, Beijing, China, with the same primers used in the PCR reactions. Species were identified by sequence comparison with accessions in the NCBI databases using the BLAST program.

### 2.4. Phylogenetic Analyses

Although ITS is an important marker used as a barcode for fungal species, it can be difficult to align ITS sequences for many groups of fungi, including *Sidera* [1,2]. Therefore, we used the most stable and conservative portion of ITS (5.8S) and the partial LSU (a fragment of about 1400 bp of the LSU) to analyze the phylogenetic relationship of the *Sidera* species. Phylogenetic analyses were performed with the Maximum Parsimony (MP), Maximum Likelihood (ML), and Bayesian Inference (BI) methods. Sequences of the species and strains were primarily adopted from 5.8S-based and 28S-based tree topologies as described by Liu et al. [2]. New sequences generated in this study, along with reference sequences retrieved from GenBank (Table 2), were aligned by MAFFT 7 ([9]; http://mafft.cbrc.jp/alignment/server/, accessed on 7 March 2022) using the “G-INS-i” strategy and manually adjusted in BioEdit [10]. Unreliably aligned sections were removed before the analyses, and efforts were made to manually inspect and improve the alignment. The data matrix was edited in Mesquite v3.70 [11]. Sequences of *Skvortzovia furfurella* (Bres.) Bononi & Hjortstam and *S. furfuracea* (Bres.) G. Gruhn & Hallenberg obtained from GenBank were used as outgroups to root the trees in 5.8S + nLSU analysis.

Maximum Parsimony analysis was applied to the 5.8S + nLSU dataset sequences. The approaches to phylogenetic analysis utilized those conducted by Chen and Cui [12], and the tree was constructed using PAUP* version 4.0 beta 10 [13]. All the characters were equally weighted, and gaps were treated as missing data. Trees were inferred using the heuristic search option with tree bisection and reconnection (TBR) branch swapping, and 1000 random sequence addition maxtrees were set to 5000. Branches of zero length were collapsed, and all the parsimonious trees were saved. Clade robustness was assessed using a bootstrap (BT) analysis with 1000 replicates [14]. Descriptive tree statistics, including the Consistency Index (CI), Homoplasy Index (HI), Rescaled Consistency index (RC), Retention Index (RI), and tree length (TL), were calculated for each Maximum Parsimonious Tree (MPT) generated. 

The research using ML was conducted using RaxML-HPC v. 8.2.3 [15] and RaxML-HPC through the CIPRES Science Gateway ([16]; http://www.phylo.org, accessed on 7 March 2022). Statistical support values (BS) were obtained using nonparametric bootstrapping with 1000 replicates. The BI analysis was performed with MrBayes 3.2.7a [17]. Four Markov chains were run for two runs from random starting trees for 1 million generations until the split deviation frequency value <0.01, and the trees were sampled at every 1000th generation. The first 25% of the sampled trees were discarded as burn-in, and the remaining ones were used to reconstruct a majority rule consensus tree and calculate the Bayesian Posterior Probabilities (BPP) of the clades. 

A total of 24 models of evolution were scored using PAUP* version 4.0 beta 10 [13]. Optimal substitution models for the combined dataset were then determined using the Akaike Information Criterion (AIC) implemented in MrModeltest 2.3 [18,19]. The model GTR + I + G was selected for use in the Maximum Likelihood (ML) and Bayesian Inference (BI) analyses.

Branches that received bootstrap support for Maximum Likelihood (BS), Maximum Parsimony (BP), and Bayesian Posterior Probabilities (BPP) >70% (BS), 70% (BP), and 0.95 (BPP) were considered to be significantly supported. In addition, the ML analysis resulted in the best tree, and only the ML tree is shown along with the support values from the MP and BI analyses. FigTree v1.4.4 [20] was used to visualize the resulting tree.

## 3. Results

### 3.1. Phylogenetic Analyses

The concatenated 5.8S + nLSU dataset contained sequences from 27 fungal specimens that represented 14 taxa of *Sidera* (two are treated as *S. vulgaris* sensu lato) (Table 2). The dataset had an aligned length of 1514 characters, with 1334 characters that were constant, 57 that were variable but parsimony-uninformative, and 123 that were parsimony-informative. The MP analysis yielded three equally parsimonious trees (TL = 341, CI = 0.607, RI = 0.790, RC = 0.479, and HI = 0.393). In addition, the average standard deviation of the split frequencies was 0.007886 (BI). 

**Figure 1:**
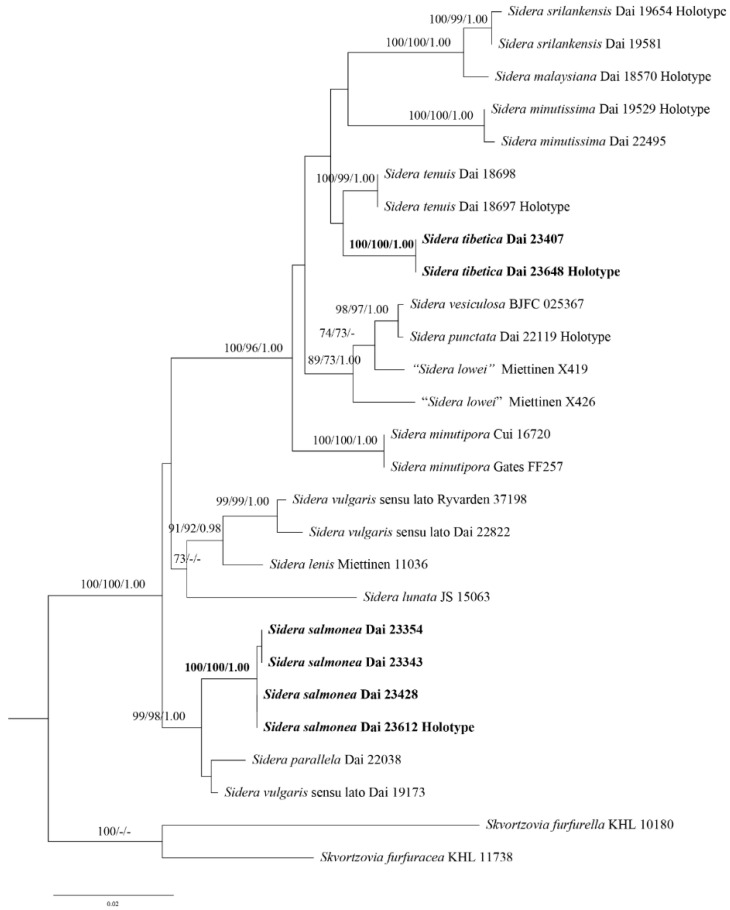
Phylogeny of *Sidera* and related species generated by ML analyses based on combined 5.8S + nLSU sequences. Branches are labelled with ML bootstrap >70%, parsimony bootstrap proportions >70%, and Bayesian Posterior Probabilities >0.95. New species are indicated in bold. ML, Maximum Likelihood; nLSU, nuclear large subunit RNA.

The phylogeny inferred from the 5.8S + nLSU sequences (Figure 1) demonstrated that the new species *S. salmonea* and *S. tibetica* clustered in the *Sidera* clade. *Sidera salmonea* grouped with *S. parallela* Y.C. Dai et al. and *S.* *vulgaris* sensu lato (Dai 19173) with strong support (99% BS, 98% BP, and 1.00 BPP). In contrast, *S. tibetica* grouped with *S. tenuis* Y.C. Dai et al. with weak support (35% BS and 0.63 BPP).

### 3.2. Taxonomy

**Figure 2:**
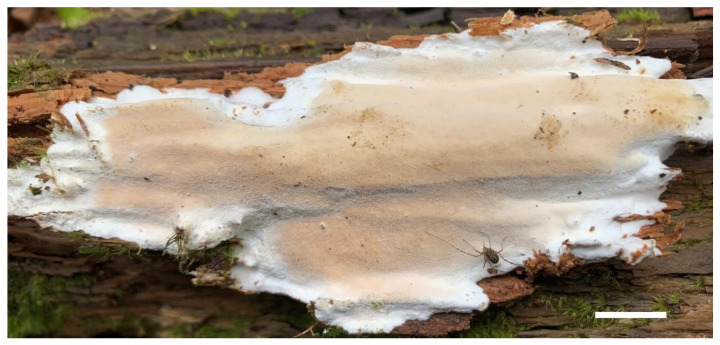
A basidioma of *Sidera salmonea* (Paratype, Dai 23354). Scale bar = 1.0 cm. Photo by Yu-Cheng Dai.

**Figure 3:**
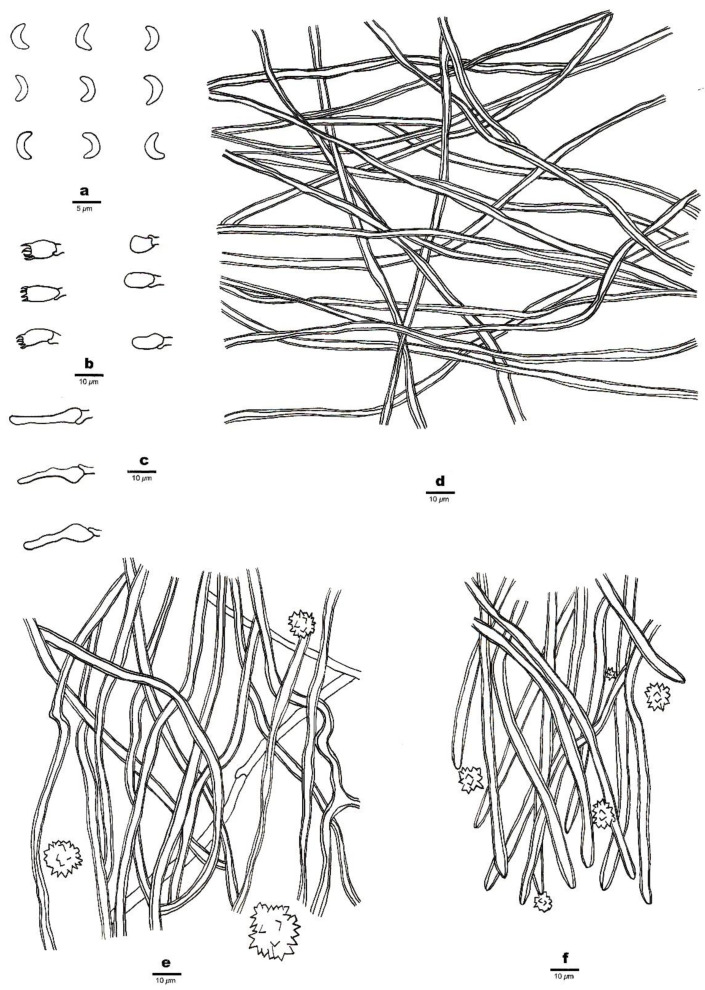
Microscopic structures of *Sidera salmonea* (Holotype, Dai 23612). (**a**) Basidiospores. (**b**) Basidia and basidioles. (**c**) Cystidioles. (**d**) Hyphae from the subiculum. (**e**) Hyphae from the trama. (**f**) Hyphae at the dissepiment edge. Drawings by Zhan-Bo Liu.

1. ***Sidera salmonea*** Z.B. Liu, J. Yu & F. Wu, sp. Nov. (Figure 2 and Figure 3)

MycoBank number: MB 843515.

Diagnosis—Annual, resupinate basidioma, salmon pores with a distinctly white margin, angular pores (7–9 per mm), a dimitic hyphal system, and lunate basidiospores 3–3.5 × 0.9–1.1 μm.

Etymology—*Salmonea* (Lat.): refers to salmon fresh pores produced by the species.

Type—China, Tibet, Bomi County, on rotten wood of *Picea*, 26 October, 2021, Dai 23612 (holotype BJFC 038184).

Basidioma—Annual, resupinate, soft corky when fresh and dry, up to 10 cm long, 6 cm wide, and 12 mm thick at the center; pore surface locally verruculose, salmon (6A4) and slightly shiny when fresh and dry, uncracked when fresh or occasionally cracked when dry; sterile margin distinct, fimbriate, white; pores angular, 7–9 per mm; dissepiments thin to thick, entire; subiculum white, cottony and up to 5 mm thick; tubes white to salmon (6A4), up to 7 mm long.

Hyphal structure—Hyphal system dimitic; generative hyphae bearing clamp connections; skeletal hyphae dominant; all hyphae IKI–, CB–; tissue unchanged in KOH.

Subiculum—Generative hyphae infrequent in the subiculum; skeletal hyphae dominant, thick-walled with a wide lumen, interwoven, rarely branched, 1–3 μm in diameter; rosette-like crystals absent.

Tubes—Generative hyphae occasionally present, thin-walled, hyaline, rarely branched, 1.5–2 μm in diameter; skeletal hyphae thick-walled with a wide lumen, interwoven, occasionally branched, 1–3 μm in diameter, dominating in trama and at dissepiment edges; rosette-like crystals abundant in tube trama and dissepiment edges, 3–30 μm in diameter; cystidia absent; cystidioles present, fusoid, hyaline, thin-walled, basally swollen, with a sharp or often hyphoid neck, 15.5–16.5 × 3.5–4 μm; basidia barrel-shaped, hyaline, bearing four sterigmata and a basal clamp connection, 6–8 × 3.5–4 μm; basidioles that were similar in shape to basidia but were slightly shorter.

Basidiospores—Lunate, smooth, thin-walled, hyaline, occasionally with one or more guttules, IKI–, CB–, 3–3.5 (–3.8) × (0.8–) 0.9–1.1 (–1.3) μm, L = 3.23 μm, W = 1.04 μm, Q = 3.03–3.21 (n = 60/2).

Additional specimens (paratypes) examined—China, Tibet, Sejila Mountain, on charred roots of *Abies*, 23 October 2021, Dai 23343 (BJFC 037914); on rotten wood of *Abies*, 23.X.2021, Dai 23354 (BJFC 037925); on rotten wood of Chinese white pine (*Pinus armandii*), 24 October 2021, Dai 23428 (BJFC 038000).

**Figure 4:**
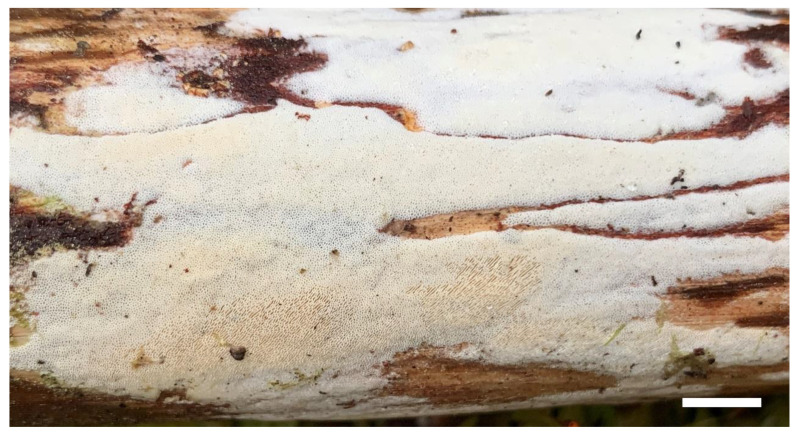
A basidioma of *Sidera tibetica* (Holotype, Dai 23648). Scale bar = 1.0 cm. Photo by Yu-Cheng Dai.

**Figure 5:**
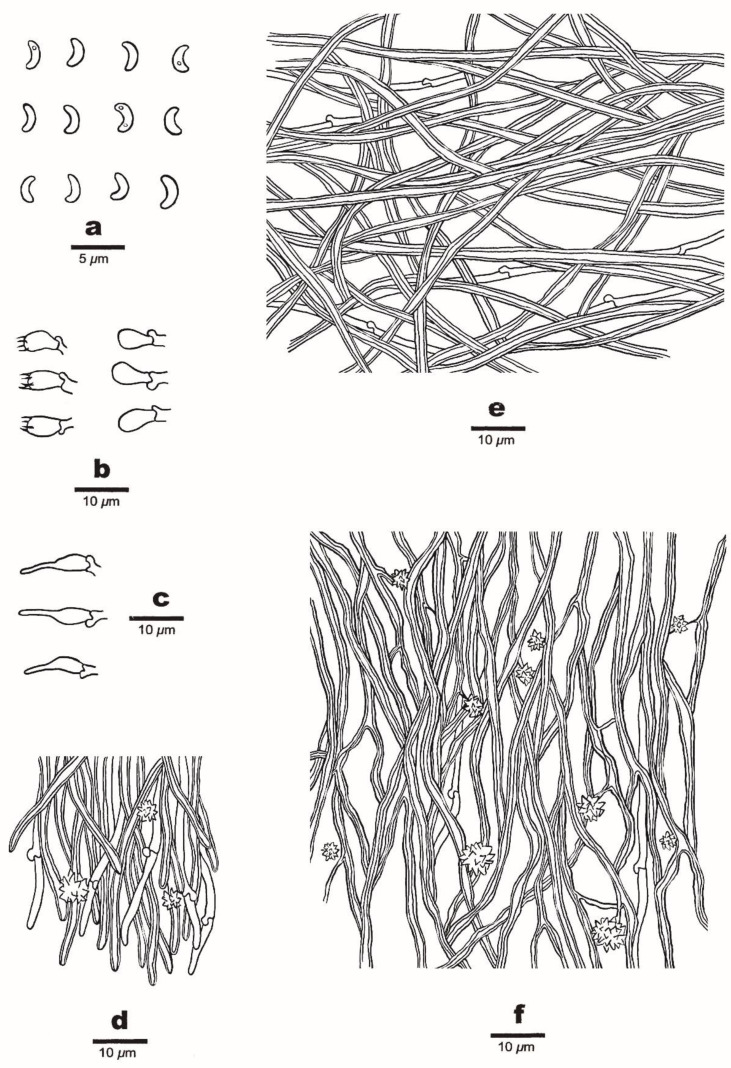
Microscopic structures of *Sidera tibetica* (Holotype, Dai 23648). (**a**) Basidiospores. (**b**) Basidia and basidioles. (**c**) Cystidioles. (**d**) Hyphae at the dissepiment edge. (**e**) Hyphae from the subiculum. (**f**) Hyphae from the trama. Drawings by Meng Zhou.

2. ***Sidera tibetica*** Z.B. Liu, J. Yu & F. Wu, sp. Nov. Figure 4 and Figure 5

MycoBank number: MB 843516.

Diagnosis—Annual, resupinate basidioma with a white to cream fresh pore surface that becomes cream to honey-yellow and shiny upon drying, a dimitic hyphal system, round pores (7–8 per mm), and lunate basidiospores 2.9–3.1 × 1–1.1 μm.

Etymology—*Tibetica* (Lat.): referring to the distribution of species in Tibet.

Type—China, Tibet, Bomi County, Gangcun Spruce Park, on a rotten branch of Chinese white pine (*Pinus armandii*), 27 October 2021, Dai 23648 (holotype BJFC 038220).

Basidioma—Annual, resupinate, soft corky when fresh and dry, 4 cm wide, up to 10 cm long, and <0.8 mm thick at the center; pore surface white to cream when fresh, becoming cream to honey yellow and shiny when dry; sterile margin indistinct, cottony, white, thinning out; pores round, 7–8 per mm; dissepiments thin, entire to slightly lacerate; subiculum white, up to 0.1 mm thick; tubes concolorous with a poroid surface, up to 0.7 mm long.

Hyphal structure—Hyphal system dimitic; generative hyphae bearing clamp connections; skeletal hyphae dominant; all hyphae IKI–, CB–; tissue unchanged in KOH.

Subiculum—Generative hyphae infrequent, thin-walled, hyaline, rarely branched, 1–3 μm in diameter; skeletal hyphae dominant, interwoven, unbranched, 2–4 μm diameter.

Tubes—Generative hyphae infrequent, thin-walled, hyaline, rarely branched, 1–3 μm in diameter; skeletal hyphae dominant, thick-walled with a wide to medium lumen, hyaline, frequently branched, interwoven, flexuous, 2–4 μm in diameter; rosette-like crystals frequently present; cystidia absent; cystidioles present, fusoid, thin-walled, hyaline, basally swollen, with hyphoid neck and sharp tip, 13–15 × 3–4 μm; basidia barrel-shaped, hyaline, bearing four sterigmata and a basal clamp connection, 8–9.5 × 3.5–4.5 μm; basidioles pyriform, shorter than the basidia.

Basidiospores—Lunate, thin-walled, hyaline, smooth, occasionally with one or two guttules, IKI–, CB–, (2.8–) 2.9–3.1 (–3.3) × 1–1.1 (–1.2) μm, L = 3.01 μm, W = 1.05 μm, Q = 2.78–2.91 (n = 90/3).

Additional specimens (paratypes) examined—China, Tibet, Bomi County, Yigong, on a rotten branch of *Pinus armandii*, 24 October 2021, Dai 23407 (BJFC 037979); on rotten wood of Yunnan pine (*Pinus yunnanensis*), 24 October 2021, Dai 23486 (BJFC 038058).

                        **A key to species of *Sidera* in worldwide**


1. Hymenium grandinioid to odontioid
*S. lunata*
1. Hymenium poroid22. Hyphal system monomitic32. Hyphal system dimitic63. Basidiospores mostly < 1 μm in width43. Basidiospores mostly > 1 μm in width54. Pores 7–9 per mm; basidiospores 2.9–3.7 μm long
*S. vesiculosa*
4. Pores 6–7 per mm; basidiospores 3.9–4.5 μm long
*S. roseo-bubalina*
5. Pores 6–8 per mm; cystidioles present, some branched 
*S. lowei*
5. Pores 8–9 per mm; cystidioles absent
*S.*
*punctata*
6. Basidiospores > 1.5 μm in width
*S. lenis*
6. Basidiospores < 1.5 μm in width77. Skeletal hyphae becoming swollen in KOH87. Skeletal hyphae almost unchanged in KOH108. Pores 5–7 per mm; basidiospores 3.7–4.3 μm long
*S. minutipora*
8. Pores 9–11 per mm; basidiospores 2.9–3.3 μm long99. Basidiospores allantoid, skeletal hyphae distinctly swollen in KOH
*S. inflata*
9. Basidiospores lunate, skeletal hyphae slightly swollen in KOH
*S. malaysiana*
10. Tramal hyphae parallel along the tubes
*S. parallela*
10. Tramal hyphae interwoven1111. Generative hyphae at dissepiments even 1211. Generative hyphae at dissepiments with swollen tips1412. Basidiospores > 3.5 μm long
*S. srilankensis*
12. Basidiospores < 3.5 μm long1313. Sterile margin distinct, white; basidiospore length/width > 3
*S. salmonea*
13. Sterile margin indistinct to almost absent; basidiospore length/width < 3
*S. tibetica*
14. Basidiospores < 3.6 μm long
*S. vulgaris*
14. Basidiospores > 3.8 μm long1515. Sterile margin distinct, fimbriate; basidiospore length/width < 4
*S. minutissima*
15. Sterile margin indistinct to almost absent; basidiospore length/width > 4
*S. tenuis*



## 4. Discussion

Two new species, *S. salmonea,* and *S. tibetica*, are described in this study based on morphological characters and phylogenetic analyses. Phylogenetically, *S. salmonea* and *S. tibetica* are nested in the *Sidera* clade based on the 5.8S + nLSU sequence data (Figure 1). *Sidera salmonea* grouped with *S. parallela* and *S. vulgaris* sensu lato (Dai 19173) with strong support (99% BS, 98% BP, and 1.00 BPP). *Sidera salmonea* can be morphologically distinguished from *S. parallela* by its interwoven tramal hyphae, smaller pores (7–9 per mm vs. 6–8 per mm, [21]), and longer basidiospores (3–3.5 μm vs. 2.8–3.3 μm, [21]). In addition, *S. salmonea* grows on rotten gymnosperm wood, while *S. parallela* grows on rotten angiosperm wood [21]. *Sidera salmonea* differs from *S. vulgaris* sensu lato (Dai 19173) owing to its larger pores (7–9 per mm vs. 9–11 per mm) and shorter basidiospores (3–3.5 μm vs. 3.5–4.2 μm). This phylogenetic analysis indicated that two specimens of *S. tibetica* formed a lineage with strong support (100% BP, 100% BS, and 1.00 BPP) (Figure 1) and grouped with *S. tenuis* with weak support (35% BS and 0.63 BPP). *Sidera tibetica* differs from *S. tenuis* by its larger pores (7–8 per mm vs. 8–10 per mm, [21]) and shorter basidiospores (2.9–3.1 μm vs. 4.2–5 μm, [21]). In addition, *S. tenuis* grows on *Eucalyptus* [21], while *S. tibetica* grows on *Pinus*. 

Species in the genus *Sidera* are lignicolous and cause a white rot. They are found in North and South America, Eurasia, Africa, Australia, and New Zealand, in boreal, temperate, and tropical climates [1]. Two new species were collected from Tibet. Tibet has the most important virgin forests in southwest China, and such forests (typical boreal natural forests) provide favorable environments for wood-decaying fungi. Numerous new species have been found in Tibet [6,22,23,24,25,26,27,28,29], and this paper confirms that the diversity of fungi is very rich in the montane forests of the Eastern Himalayas.

## Figures and Tables

**Table 1:** The main characteristics of *Sidera* species. Pore and basidiospore sizes mainly from Liu et al. [2].

Species	Growing Habit	Hymenophore	Hyphal System	Cystidioles	Skeletal Hyphae in KOH	Spores Shape	Spore Size (µm)
*S. inflata*	Annual	Poroid, 9–10/mm	Dimitic	Present	Swollen	Allantoid	3–3.3 × 0.9–1.1
*S. lenis*	Perennial	Poroid, 4–6/mm	Dimitic	Present	Swollen	Allantoid to lunate	3.9–4.9 × 1.5–2
*S. lowei*	Annual	Poroid, 6–8/mm	Monomitic	Present, some branched	–	Allantoid	3.5–5 × 1–1.2
*S. lunata*	Annual	Hydnoid, 8–9/mm	Monomitic	Present	–	Allantoid	2.5–3.8 × 1.6–1.9
*S. malaysiana*	Annual	Poroid, 9–11/mm	Dimitic	Present	Swollen	Lunate	2.9–3.2 × 1–1.2
*S. minutipora*	Annual	Poroid, 5–7/mm	Dimitic	Present	Swollen	Allantoid	3.7–4.3 × 1–1.3
*S. minutissima*	Annual	Poroid, 7–9/mm	Dimitic	Present	Almost unchanged	Allantoid	3.8–4.4 × 0.9–1.3
*S. parallela*	Annual	Poroid, 6–8/mm	Dimitic	Present	Almost unchanged	Lunate	2.8–3.3 × 0.9–1.2
*S. punctata*	Annual	Poroid, 8–9/mm	Monomitic	Absent	–	Allantoid to lunate	3.8–4.8 × 1–1.3
*S. roseo-bubalina*	Annual	Poroid, 6–7/mm	Monomitic	Present	–	Lunate	3.9–4.5 × 0.8–1
***S. salmonea***	**Annual**	**Poroid, 7–9/mm**	**Dimitic**	**Present**	**Almost unchanged**	**Lunate**	**3–3.5 × 0.9–1.1**
*S. srilankensis*	Annual	Poroid, 6–8/mm	Dimitic	Present	Almost unchanged	Lunate	3.5–4 × 1–1.3
***S. tibetica***	**Annual**	**Poroid, 7–8/mm**	**Dimitic**	**Present**	**Almost unchanged**	**Lunate**	**2.9–3.1 × 1–1.1**
*S. tenuis*	Annual	Poroid, 8–10/mm	Dimitic	Present	Almost unchanged	Allantoid	4.2–5 × 0.8–1
*S. vesiculosa*	Annual	Poroid, 7–9/mm	Monomitic	Present	–	Allantoid to lunate	2.9–3.7 × 0.6–1
*S. vulgaris*	Perennial	Poroid, 6–8/mm	Dimitic	Present, some branched	Almost unchanged	Allantoid to lunate	2.9–3.6 × 0.9–1.4

New species are shown in bold.

**Table 2:** Taxa information and GenBank accession numbers of the sequences used in this study.

Species	Specimen No.	Locality	GenBank Accession No.	
			ITS	nLSU
*S. lenis*	Miettinen 11036	Finland	FN907914	FN907914
“*S. lowei*”	Miettinen X419	Venezuela	FN907917	FN907917
“*S. lowei*”	Miettinen X426	New Zealand	FN907919	FN907919
*S. lunata*	JS 15063	Norway	DQ873593	DQ873593
*S. malaysiana*	Dai 18570	Malaysia	MW198481	MW192007
*S. minutipora*	Gates FF257	Australia	FN907922	FN907922
*S. minutipora*	Cui 16720	Australia	MN621349	MN621348
*S. minutissima*	Dai 19529	Sri Lanka	MN621352	MN621350
*S. minutissima*	Dai 22495	China	OM974248 *	OM974240 *
*S. parallela*	Dai 22038	China	MW477793 *	MW474964 *
*S. punctata*	Dai 22119	China	MW418438	MW418437
***S. salmonea***	**Dai 23343**	**China**	OM974249 *	OM974241 *
***S. salmonea***	**Dai 23354**	**China**	OM974250 *	OM974242 *
***S. salmonea***	**Dai 23428**	**China**	OM974251 *	OM974243 *
***S. salmonea***	**Dai 23612**	**China**	**–**	OM974247 *
*S. srilankensis*	Dai 19581	Sri Lanka	MN621345	MN621347
*S. srilankensis*	Dai 19654	Sri Lanka	MN621344	MN621346
*S. tenuis*	Dai 18697	Australia	MK331865	MK331867
*S. tenuis*	Dai 18698	Australia	MK331866	MK331868
***S. tibetica***	**Dai 23407**	**China**	OM974252 *	OM974244 *
***S. tibetica***	**Dai 23648**	**China**	OM974253 *	OM974245 *
*S. vesiculosa*	BJFC025367	Singapore	MH636565	MH636567
*S. vulgaris* sensu lato	Ryvarden 37198	New Zealand	FN907918	FN907918
*S. vulgaris* sensu lato	Dai 19173	Canada	MW198477 *	MW192005 *
*S. vulgaris* sensu lato	Dai 22822	China	OM974254 *	OM974246 *
*Skvortzovia furfuracea*	KHL 11738	Finland	DQ873648	DQ873648
*S. furfurella*	KHL 10180	Puerto Rico	DQ873649	DQ873649

* Newly generated sequences for this study. New species are shown in bold.

## Data Availability

The sequence alignment was deposited at TreeBase (submission ID 29499; http://purl.org/phylo/treebase/phylows/study/TB2:S29499?x-access-code=770f9dfc79ea7f489fbf2fc56e55ec7e&format=html accessed on 7 March 2022).
